# A Deformable Configuration Planning Framework for a Parallel Wheel-Legged Robot Equipped with Lidar

**DOI:** 10.3390/s20195614

**Published:** 2020-10-01

**Authors:** Fei Guo, Shoukun Wang, Binkai Yue, Junzheng Wang

**Affiliations:** 1 School of Automation, Beijing Institute of Technology, No. 5 South Zhongguancun Street, Haidian District, Beijing 100081, China; guofei@bit.edu.cn (F.G.); binkay@126.com (B.Y.); wangjz@bit.edu.cn (J.W.); 2 State Key Laboratory of Intelligent Control and Decision of Complex System at School of Automation, Beijing Institute of Technology, Beijing 100081, China; 3 Key Laboratory Ministry of Industry and Information Technology, Beijing Institute of Technology, No. 5 South Zhongguancun Street, Haidian District, Beijing 100081, China

**Keywords:** wheel-legged hybrid robot, trajectory optimization, motion planning, obstacle negotiation, parallel mechanism

## Abstract

The wheel-legged hybrid robot (WLHR) is capable of adapting height and wheelbase configuration to traverse obstacles or rolling in confined space. Compared with legged and wheeled machines, it can be applied for more challenging mobile robotic exercises using the enhanced environment adapting performance. To make full use of the deformability and traversability of WHLR with parallel Stewart mechanism, this paper presents an optimization-driven planning framework for WHLR with parallel Stewart mechanism by abstracting the robot as a deformable bounding box. It will improve the obstacle negotiation ability of the high degree-of-freedoms robot, resulting in a shorter path through adjusting wheelbase of support polygon or trunk height instead of using a fixed configuration for wheeled robots. In the planning framework, we firstly proposed a pre-calculated signed distance field (SDF) mapping method based on point cloud data collected from a lidar sensor and a KD -tree-based point cloud fusion approach. Then, a covariant gradient optimization method is presented, which generates smooth, deformable-configuration, as well as collision-free trajectories in confined narrow spaces. Finally, with the user-defined driving velocity and position as motion inputs, obstacle-avoidancing actions including expanding or shrinking foothold polygon and lifting trunk were effectively testified in realistic conditions, demonstrating the practicability of our methodology. We analyzed the success rate of proposed framework in four different terrain scenarios through deforming configuration rather than bypassing obstacles.

## 1. Introduction

The legged machine shows its excellent adaptability of negotiating the convex obstacles. However, these actions necessitate substantial planning calculation [1], complicated model to sustain walking stability while swinging foot [2,3], and obstacle with a flat surface and relatively low height to maintain foothold safety [4]. As legged robots have been developing to become popular, there is growing interest in incorporating benefits from the wheeled and legged mechanism to realize hybrid locomotion, intensifying the intention of boosting mobility and efficiency. Several wheel-legged hybrid robots (WLHRs) have shown their ability of adapting to convex environment. The robot CENTAURO [5] which are equipped with steerable wheels on legs’ ending allows for executing steps, omnidirectional steering, and dominating a large variety of mobile manipulation missions, demonstrating its various moving modes in different types of terrains.

Within the correlative application territory, obstacle negotiation has extensively involved several aspects among mobile robots, under the priority optimizing the robot stability and trajectory smoothness. With sustaining walking stability and kinematic feasibility, Yue [6] adopted the concept of artificial potential field on a hexapod robot to avoid obstacles. A novel obstacle avoidance method [7], Follow the Gap Method, eliminated local minimum problem in path planning on an autonomous ground vehicle with Ackermann steering geometry. The collision-free paths [8] were planned using probabilistic roadmaps targeting on a given location. A laser-based people tracking component estimated the motions of humans. Guaranteeing smoothness and collision-free, a reciprocal orientation algorithm [9] planned trajectories for multi-robot without direct communication with other robots. With the assumption of flat terrain, an optimal global path was planned [10]. Connecting states through local trajectory generator is set up as a graph-search problem, which is solved by best-first search. Furthermore, a precise tracking controller is also important for underactuated systems especially with unknown parameters and disturbances. Adaptive neural networks [11] approximated unknown dynamics and updated parameters to decrease tracking error.

To autonomously drive in different environment, specific terrain description can effectively enhance robot shifting availability. The hierarchical data structure [12] identified and stored obstacle cells as non-uniform $2^{m}$ trees, which is crucial before planning. The surface normals [13] on robot-centric elevation map are employed to select feasible footholds, organizing collision-free trajectories for swing feet. The planar laser scan, stereo vision, and preoperative sensing mounted on BigDog robot transformed data collection into a 2D cost representation. BigDog [14] equipped with a combination of planar laser scans, stereo vision, and preoperative sensing to perceive obstacles and placed their data collection into a 2D cost representation. Then it planed paths through a variation classic A* search and steered four legs to follow them. The Messor II hexapod [15] employed RGB-D data to combine OctoMap and elevation grid into a 3-D semantic labeling of natural environments, conducting its motion planning. Establishing a bridge between discrete and continuous planning primitively [16] leveraged each individual configuration of ground contact points (footholds) in legged locomotion which are explored to forge specified motion sequences to avoid collision without compromising COM agility. However, none of these approaches concentrated on obstacle avoidance for robot body and reconfigured relationship between limbs, which is independent in narrow space.

As for the planning algorithm for configurable robots or executing obstacle negotiation through changing arrangement between different robot components, have been implemented in several kinds of robots. Combining with point clouds segmentation and traversability analysis, an autonomous 3D path planning [17] executed rolling motion using a tracked reconfigurable vehicles on stairway and slope. Maximising the height of sensor payload, stability [18] and moving capability [19] originated from A* search to enhance on uneven terrain. Anh [20] advanced an efficient coverage path planning in a novel self-reconfigurable cleaning robot. A modified A-Star zigzag global planner can generate waypoints for the purpose of maximizing robot’s coverage area. A reconfigurable snake-like robot [21] integrated path and motion planning approach to move in challenging environments populated with obstacles like stairs. The terrain information used for motion planning is presented by a multi-resolution map. Then an extended RRT* is utilized to form a multi-layered planning model, generating a multi-stepped planning process. In Reference [22], a contact dynamic rodmaps is constructed, which generates discretized motions in each leg’s workspaces offline. Then a mapping method is designed to obtain motions in its configuration space. In the online phase, the planner can adapt to environment rapidly and generate collision-free foothold positions. In Reference [23], to acquire multifaceted navigation skill such as body slimming or lifting for obstacle avoidance, an end-to-end planning method based on deep reinforcement learning is presented, which generates motor commands directly from the height map. Furthermore, a multi-modal PRM is proposed in Reference [24] for planning problems with finite number of intersecting manifolds. It applied to determine support polygons and configurations of ATHLETE and HRP2 robot walking on flat, stairs, and undulating terrains. The convergence of an incremental variant of PRM was also proved. A wheel-legged hybrid locomotion was also executed on the ATHLETE robot simulation, but the calculation quantity of mentioned planner is considerably abundant when obstacle avoidance did not involve. The pattern of wheel-legged hybrid locomotion involves not only the driving-stepping but also the foothold polygon adaptive locomotion. MOMARO robot parameterized the stepping and driving locomotion into a single ARA* planner [25] and combined three-level abstract traversing manoeuvres [26] on stairs. Furthermore, the Value Iteration Networks (VINs) [27] planed omnidirectional driving for support polygon obstacle avoidance in cluttered terrain that combined multiple levels of abstraction. The computational cost of these planner are expensive or highly dependented on number of robot joints or offline training.

Intuitively, the obstacle negotiation issue for robot in confined space can be simplified as a deformable bounding box abstraction of robot model, which is inspired from reconfigurable and soft robots. The Randomized Possibility Graphs [28] combined the bounding boxes attached to biped robot to rapidly explore the possible actions in a variety of semi-unstructured environments. The nested robot [29] was utilized to gradually induce number of abstract models, which is called the Quotient-space roadMap Planner to search action graph until a valid path had been found. A hexapod robot Weaver [30] has the ability to adapt walking posture to tunnel environment, in which the scheduled method smoothed trajectories by Covariant Hamiltonian Optimization for Motion Planning (CHOMP) [31] for Efficient Motion Planning. The CENTAURO robot [32] transformed the Octomap into 2D occupation map to determine robot polygon shrinking and expansion to go over obstacles through traditional A* search program.

WLHR inherits the compounded superiority, including flexible obstacle-crossing function, efficiency, and agility from legged and wheeled systems. Compared to common serial leg mechanism, Stewart parallel counterpart holds higher load capacity and permits six-dimensional movability in the narrower zone, which generally creates omnidirectional movement and adjustable volume deformation on a rigid and mix-and-match manner of wheels and feet robot. There have been some researches concerning grid-searched algorithm for stepping-driving hybrid locomotion [25,26] and changeable foothold polygon [32] on two-dimensional planning to traverse obstacles, but no optimization-based composition for WLHR in three-dimensional obstacle avoidance. The BIT-NAZA robot is able to roll towards arbitrary orientation without rotating body configuration as well as adaptable trunk height and foothold polygon, which is immensely suitable constituting a rigid deformable robot. The covariant gradient optimization devotes to identify a collision-free trajectory, even when the initial trajectory contained many collisions. Actually, the proposed strategy initiates BIT-NAZA WLHR to change the wheel-legged configuration, bringing different kinds of foothold polygon into existence while in a rolling motion. To our best knowledge, this is the first time that incorporates bounding box abstraction and covariant gradient optimization algorithm to design a numerical solver on a rigid object which is also a WLHR regarded as a deformable robot. In this case, the robot is capable of independently navigating in confined spaces via morphing configuration, negotiating certain obstacles. We suppress crash between each element on the morphing geometry and obstacles in confined space since whether the crash can happen relates to the signed distance field (SDF) whose array is calculated from a robot-centric obstacle information. Succinctly, our main contribution can be enumerated as follows:

- Present and fabricate a novel WLHR prototype with Stewart parallel mechanism. The parallel structure enable robot to afford more than 300 kg burden. The full software and hardware layouts are exhibited from an individual electric motor controller to perception sensor mapper, contributing to expose how the intricate system work from low to high level.
- Calculate the signed distance filed from robot-centric obstacle dimension box, which is collect from lidar after filter the ground point cloud. The incremental linear fitting method and KD tree clustering approach were adopted to describe width and height of obstacle box.
- Introduce a deformable bounding box abstraction for WLHR model, which is incorporated with SDF representing obstacles to check collision.
- Propose a covariant gradients-based trajectory optimization formulation that is applied to generate smooth and collision-free motions for a rigid deformable WLHR through changing trunk height and wheelbase. Integrate the planning framework into morphing locomotion in confined space, satisfying the kinematic reachability, smoothness, and obstacle avoidance constraints.
- Validate feasibility of our methodology on a simulated model and practical WLHR prototype in four scenes. A suite of intensive analysis and comparison for algorithm computation and robot deformable properties are displayed through line chart and histogram.

## 2. A Wheel-Legged Robot (WLHR) Description and Framework Overview

**Figure 1:**
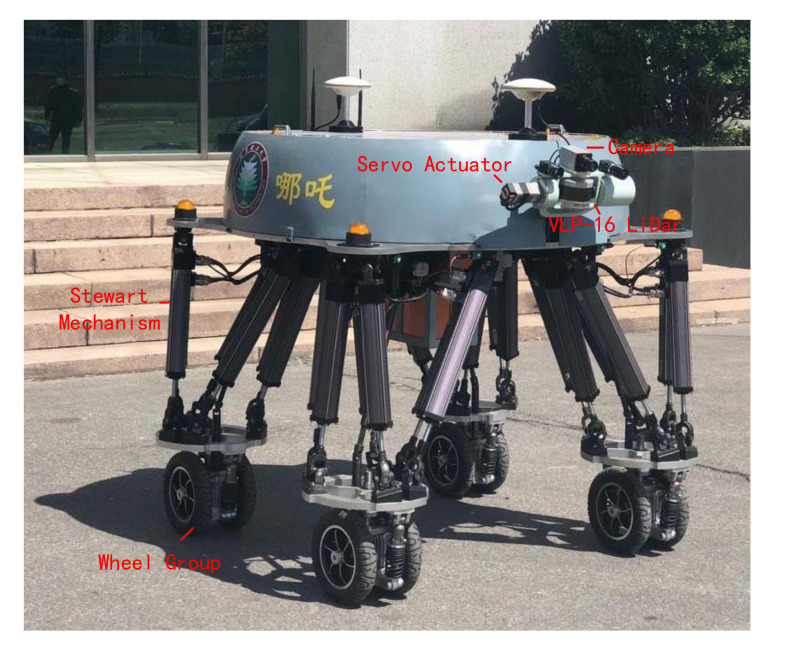
The physical prototype of BIT-NAZA robot.

The BIT-NAZA robot showed in Figure 1 is designed as a quadrupedal machine equipped with four active wheel groups at four pelmas respectively. The mechatronic structure of each leg is comprised of six stretchable linkages with electrical actuators in parallel. The parallel construction enhances the body’s payload capability and possesses six freedom of degrees, rendering the feasibility of horizontally rotating the wheel orientation. Furthermore, there are a linear displacement encoder and a force sensor internally-installed in each linkage scaling the displacement and force along stretching orientation. An inertial measurement unit (IMU) is mounted on robot torso to supply angle, angular velocity, and angular acceleration data along three axes. The robot stands approximately 1.4 m high when every linkage reaches its neutral position, as well as can maximally afford 350 kg with a total mass of about 300 kg (encompassing overall hardware, batteries and sensors).

**Figure 2:**
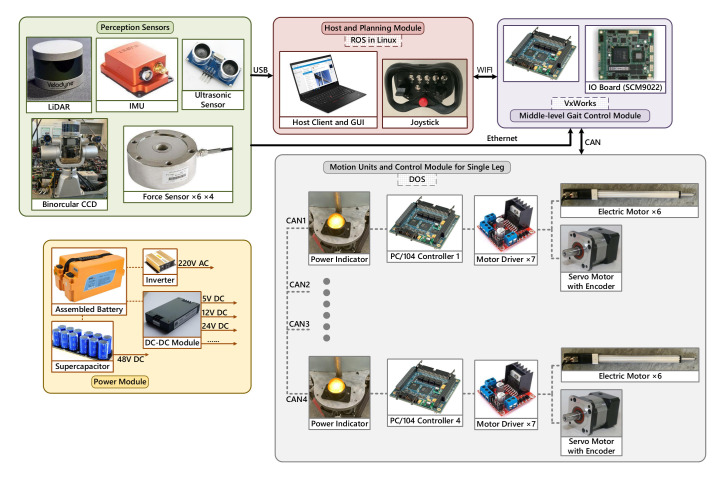
The hardware and software components of the WLHR system.

The BIT-NAZA was developed as a research platform for unmanned autonomous moving missions in challenging environments. Accounting for running on rugged terrains, the wheel-legged robot is capable of switching different types of locomotion among regular quadruped stepping, full-wheeled driving, and stepping-rolling hybrid locomotion, in which the Stewart mechanism urges wheels and robot base to change relative positions constituting a deformable robot independently. The proposed system architecture contains multiple hardware and software components embodied in Figure 2. In addition to low-level actuator and middle-level gait control module operating in DOS and VxWorks separately, the mapping data disposing and planning module leverages the program environment of ROS in Linux where the scheduled framework engaged in.

**Figure 3:**
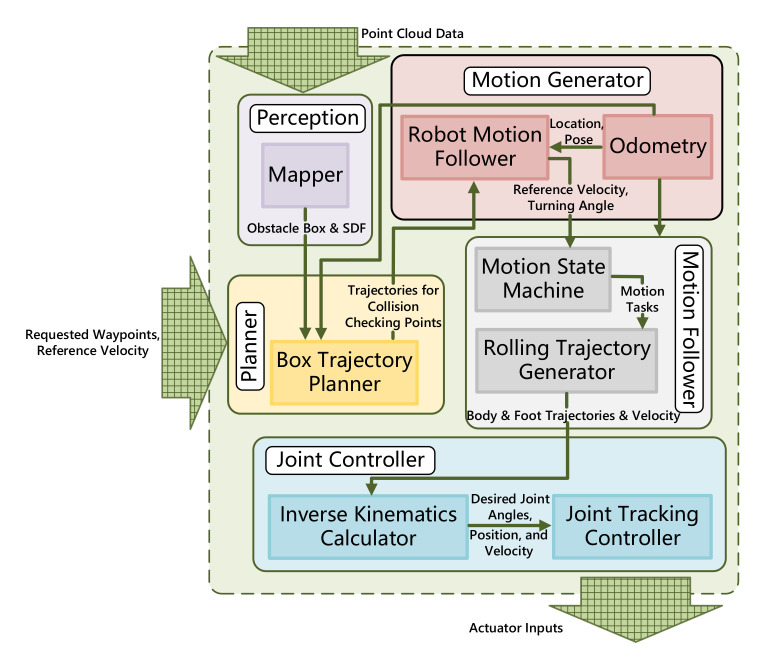
The schematic diagram of planning framework working on BIT-NAZA robot.

The block drawing scratched in Figure 3 depicts the exploited planning architecture with the input of point cloud data (PCD) collected from lidar or other depth perceptive sensors and user commands including reference velocity and requested target waypoint. The ultimate task of Planner is to plan online a pertinent sequence of trajectories for collision checking points distributed on bounding box, which allows the robot to traverse convex obstacles or pass confined space pursuing assigned waypoints. The LiDar equipped on robot accumulates point cloud data, which is transformed into width, height, and length dimension of obstacle in Mapper module. A deformable planning approach exploits covariant gradient method to prevent robot from obstacles through signed distance field, forming a smooth trajectory for bounding box abstraction. The optimal trajectories produced from planner are transformed into subsequent motion states for body and wheels in terms of current robot configuration through robot kinematic definition in Motion Generator. Additionally, wheeled odometry that accumulates the total turning angles of wheels in charge of the current location of robot and collects the trunk posture information from body-mounted IMU. The modular layer where Motion Follower formulates reactive maneuver helps us to seamlessly connect the Motion Generator and Joint Controller. The corresponding commands for different motion units are transferred and converted into joint tracking controller to follow the planned Stewart-based kinematic solution. The final output from our planning framework is actuator input containing required joint displacement, velocity and acceleration for 24 electrical cylinders and wheel motors.

## 3. Problem Formulation

Different from standard quadruped locomotion demonstrated in Reference [33], this work only concentrated on full-wheeled driving and legged-rolling hybrid locomotion to follow rough terrain variation. The whole body motion trajectories in three-dimension are executed on five action units including four wheels and body base when legs adjust space configuration between these different units with simultaneous wheeled velocity. This activity not only leads to absolute motion for robot entirety but also activates the relative motions for robot components between each other. To adapt robot to several types of terrains and avoid the high-dimensional calculation restriction, we simplify this problem through outlining a deformable bounding box which can cover the entire robot body. The box fixed on robot base would not stretch downwards to accommodate wheel-legs but can widen or narrow according to united volume of the left and right wheel-legs. The obstacle avoidance in three-dimensional confined space for a WLHR with such large quantities of actuating joints, we simplified the robot model into an bounding box in order to find smooth trajectories for different joints that satisfied several constraints. The abstract box model is convenient for finding a collision-free trajectory and in charge of width and height of whole robot, playing an important role in the framework with our trajectory definition.

### 3.1. Deformable Bounding Box Abstraction

**Figure 4:**
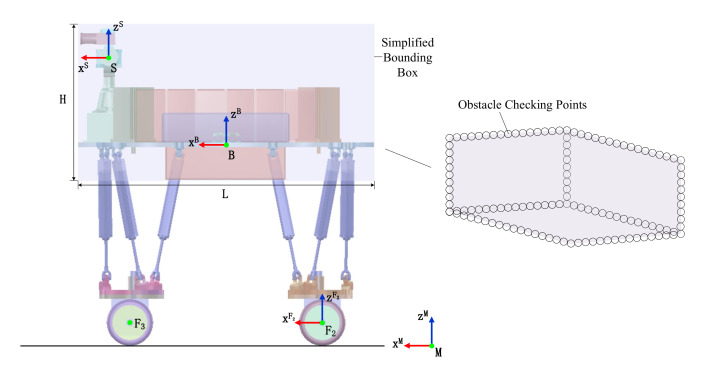
The robot model and obstacle checking points on abstract outline, which are regarded as a part of planner input.

For the purpose of avoiding obstacles, we allocate several collision checking points distributed on a bounding box $O\in R^{3}$ which outlines the robot base with constant height and length but changeable width. The abstracted geometry is attached to the body coordinate frame B as displayed in Figure 4. The simplification tremendously accelerates collision checking exploration. Entire kinds of motions are encapsulated, where the robot is able to perform while rolling in any height, direction, and width. Despite the bounding box does not encase legs, its width extending to reach the outermost points quantifies how width robot broadens. It means width of bounding box broaden or shrink its width over varying wheelbase, accounting for the lateral component of leg collision checking. If nothing in explored space collides with this geometry, the robot is ensured to be prevented from collisions.

The lateral expansion of robot *e* is prescribed as half width of the bounding box so that the box expression is (L,2·e,H) in three-dimensional space. A dense sampling distributes 10 cm interval between collision checking points on edges of the bounding box. The number of points relies on the volume and sampling density, resulting in 168 points in our case. Then we need to adopt motion sequence generator to find solutions for optimized trajectories satisfying these sufficient conditions.

### 3.2. Trajectory Definition

Once the simplified geometry is defined, the remaining theme which is whole body trajectories planning is guided in our routine. Iterating the route time and time again acquires the optimal solution, which is entailed as a series of configurations ξ at time *t* from a start configuration ξ(0) to an appointed configuration ξ(1) in the three-dimensional description.

(1) ξ(t)=[x(t),y(t),z(t),ϕ(t),e(t)]

in which *x*, *y*, and *z* compose the position expression and ϕ the yaw of the robot base *B* in map coordinate frame M, then the planner does not involve pitch and roll in this work. The bounding geometry also deforms in accordance with time *t* through the extendable width as a function of time. In practice, the trajectories for collision checking points on edges of the bounding box are separately discretized via sampling interval. Furthermore, the $i_{th}$ collision checking point can be calculated, which is relevant to the bounding box dimension definition.

(2) $$c_{i}^{B}={\left[c_{x}\cdot L,\;c_{y}\cdot e\left(z\right),\;c_{z}\cdot H\right]}^{T}$$

where coefficient $c_{x,y,z}\in \left[-1,1\right]$ uniquely represents the value how coordinate of $i_{th}$ collision checking point scales the coordinate of its corresponding vertex in body frame B, so that $c^{B}$ is also a vector expression in frame B. For instance, a collision checking point is situated at hind, left, and top vertex of bounding box, whose coefficients concerns $\left(c_{x},c_{y},c_{z}\right)=\left(-1,1,-1\right)$. Besides, the coordinate term $c_{i}^{B}$ in body frame B can be transformed into map frame M as

(3) $$c_{i}^{M}\left(\xi \right)=p_{B}^{M}+{}^{M}R_{B}\cdot c_{i}^{B}$$

where $p_{B}^{M}=\left(x,y,z\left(e\right),\phi ,e\right)$ indicates the known corresponding coordinates of collision checking point in map frame M, rotation matrix ${}^{M}R_{B}$ signifies the known transformation matrix from body frame B to map frame M. Formally, e(z) in Equation (2) views *e* as a function of *z*, since it is anticipated that the width of bounding box is able to deform as a consequence that robot lowers or lifts its trunk. Another matching expression in $p_{B}^{M}$ is written as inverse formulation.

Progressively, the respective kinematic Jacobian matrix of the $i_{th}$ collision checking point $J_{c_{i}}=\frac{\partial }{\partial \xi }c_{i}^{M}\left(\xi \right)\in R^{3\times 5}$ is inferred as

(4) $$\begin{array}{cc} J_{c_{i}} & =[I_{3\times 2},\;R\cdot \frac{\partial }{\partial z}c_{i}^{B},\frac{\partial }{\partial \phi }R\cdot c_{i}^{B},R\cdot \frac{\partial }{\partial e}c_{i}^{B}]\\ \; & =[\begin{array}{ccccc} 1 & 0 & c_{y}\cdot \sin\phi \cdot \frac{\partial e(z)}{\partial z} & -c_{x}\cdot L\cdot \sin\phi +c_{y}\cdot e\left(z\right)\cdot \cos\phi & 0\\ 0 & 1 & \cos\phi \cdot c_{y}\cdot \frac{\partial e(z)}{\partial z} & -\sin\phi \cdot c_{y}\cdot e\left(z\right)-c_{x}\cdot L\cdot \cos\phi & 0\\ 0 & 0 & 0 & 0 & \frac{\partial z(e)}{\partial e} \end{array}] \end{array}$$

with differential notations $\frac{\partial }{\partial z}c_{j}^{B}$ and $\frac{\partial }{\partial e}c_{j}^{B}$ revealing how the lateral expansion changes with *z* as well as the converse fashion. This work regards the robot as a deformable configuration among the robot body and four wheels, whose constraint depends on its body reachability [34] resulting from Stewart inverse kinematic solutions. With the consideration of this formulation, we can establish the deformable relationship between *e* and *z* as linear, scaling between the restrictive maximum and minimum limits of the robot’s workspace. Nevertheless, it could employ a more complicated mathematical description. For the purpose of guaranteeing optimizing efficiency, the work focused on a simple model which might potentially fail in a few very narrow spaces.

## 4. Obstacles Representation Collected from VLP-16 Lidar

As for the procedure of acquisition and evaluation of terrain information in this work, an on-board terrain data server enforces a Velodyne VLP-16 LiDAR sensor to collect PCD, quantitating terrain topology that is adjacent to robot. The field of view (FOV) of VLP-16 ranges 360 degrees horizontally and ±15 degrees vertically, whose available distance extends to 100 m. Additionally, the servo actuator mounted on turntable is responsible for sensor orientation adjustment to perceive farther region environment data.

After filter the ground point through multi-frame point cloud fusion and incremental linear fitting approach, the obstacle PCD is directly transformed into obstacle box information including corresponding dimension and distance from robot in map frame [35]. The topology relationship between discrete PCD is constructed by KD-Tree method. The Pairwise Linkage manages the obstacle cluster. Set a global distance $d_{c}$ to distinguish neighborhood set and other PCD sets. For each point $p_{i}$, record corresponding nearest distance in sequence $D_{c}$. Calculate cut-off distance $d_{c}$ with customized weight scale and mid-value in $D_{c}$:

(5) $$d_{c}=scale\cdot median\left(D_{c}\right)$$

The point density is calculated through recent point number and Gaussian kernel function as follows:

(6) $$\rho_{i}=\sum_{j\in [1,N],j\neq i}exp(-{\left(\frac{d_{ij}}{d_{c}}\right)}^{2})$$

in which $d_{ij}$ represents distance between point $p_{i}$ and $p_{j}$ inside epsilon neighborhood $L_{i}$. $p_{j}$ is the nearest point whose corresponding density of is larger than that of $p_{i}$ and mark that they are in a same cluster. If the density of $p_{i}$ is a local maximum, we consider $p_{i}$ as center of cluster. Project all of obstacle points on X-O-Y ground surface and X-O-Z surface model, we can compute precise width and height dimension of obstacle.

The metric which evaluates the distance from collision checking element $c_{i}\left(x,y,z\right)\in R^{3}$ to the boundary of nearest obstacle enables the function $D\left(c\right):R^{3}\to R$ to be pre-calculated and stored that employs Euclidean Transform Norm. A straightforward strategy is implemented to estimate obstacle potential in static exploring space. The SDF calculation is constructed as a pre-processing procedure before optimization to realize collision checking.

A workspace cost function $u:R^{3}\to R$ is to penalize the robot configuration q in the workspace for serving collision checking elements $c_{i}\in O$ to approach obstacles.

(7) $$u\left(c\right)=\{\begin{array}{ccc} D\left(c\right)+\frac{1}{2}\epsilon , & \; & if\;D(c)<0\\ \frac{1}{2\epsilon }{\left(D\left(c\right)-\epsilon \right)}^{2}, & \; & if\;0<D(c)\leq \epsilon \\ 0, & \; & otherwise \end{array}$$

Furthermore, we adopt the finite differencing to approximate the obstacle distance gradient $\nabla u=(\frac{\partial u}{\partial x},\frac{\partial u}{\partial y},\frac{\partial u}{\partial z})$, boosting the collision checking speed utilizing the primitives on geometric abstraction.

**Figure 5:**
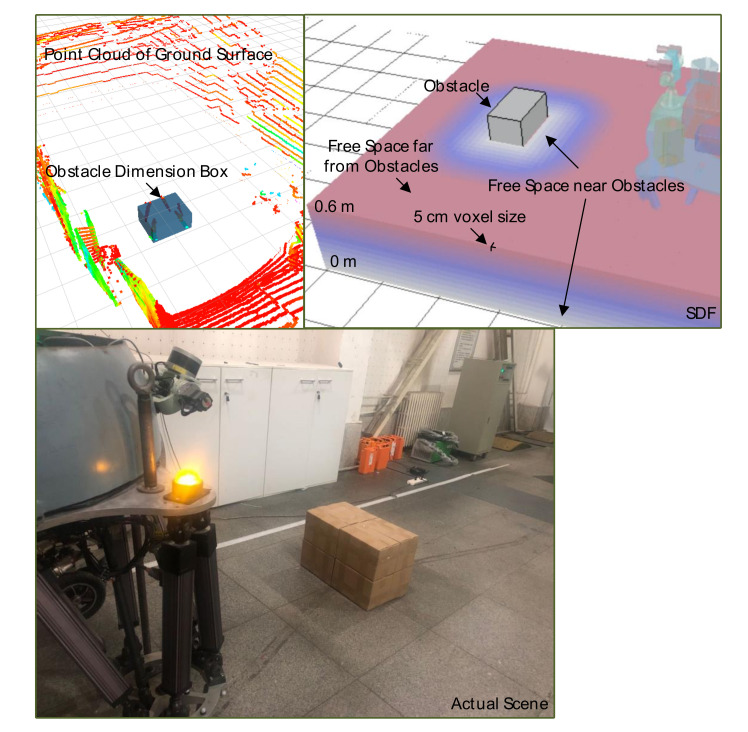
Obstacle description visualization in different transformation.

Subsequently, the SDF computation is updated with resolution in our case of 5 cm per pixel through Equaption (7), whose disposing course is displayed in Figure 5. The top figures visualized PCD collection from Velodyne VLP-16, obstacle box visualization and SDF representation that describe the environment information. A robot-centric 4 m × 3 m × 0.6 m SDF layouts cell by cell with different color following the obstacle cost function u(c) variation.

## 5. Planning and Motion Execution in Confined Space

Here we analyze how the mapping and planning module formalized in Figure 3, which operates deformable trajectory optimizing for robot obstacle negotiation in confined space. With the update rule of gradient optimization, the proposed framework produces a high-quality trajectory with three degrees of freedom which are both smooth removing unfeasible motions and collision-free. Originally, the simplified robot abstraction and SDF deriving from obstacle dimension are discretized as geometric depiction. The planner exposition conceptualizes covariant gradient descent to minimize an objective over trajectory set through workspace and joint limit constrained by robot kinematics, along with a potential which measures the amount of element configuration for trajectory containing velocity and acceleration.

### 5.1. Deformation Planning

Formally, deformable abstraction is continuous and well transformed through the Jacobian Matrix. Our numerical optimal solver is pretty appropriate for this deformable planning issue. It discretizes trajectory into a sequence of *n* waypoints ${\left[q_{1}^{T},\ldots ,q_{n}^{T}\right]}^{T}\in R^{n\times 5}$ over equal time duration Δt for robot base B. The dynamical quantities is also evaluated utilizing finite differential for corresponding collision checking elements on the bounding box. It is ascertained that the initial configuration $\xi \left(0\right)=q_{0}$ and requested waypoint $\xi \left(1\right)=q_{n+1}$ are constant once they are assigned.

(8) $$\xi_{k+1}=\xi_{k}-\frac{1}{\eta }A^{-1}\bar{\nabla }U\left[\xi_{k}\right]$$

The iterating criterion for deformable optimization integrates upgrading rule that is functional covariant gradient descent with a Hessian matrix *A* behaving as a smoothing operator and expanding gradient across trajectories. The learning rate η determines how fast or slow we will move towards the optimal solution over each iteration *k*. For what the objective functional $\bar{\nabla }U\left[\xi_{k}\right]$ measures, two complementary aspects involve encouraging trajectory smoothness and refraining trajectories close to obstacles through the prescribed amount. Two gradient terms $\bar{\nabla }F_{obs}\left[\xi_{k}\right]$ and $\bar{\nabla }F_{smooth}\left[\xi_{k}\right]$ acting on trajectory $\xi_{k}$ can be incorporated as a weighted sum.

(9) $$\bar{\nabla }U\left[\xi_{k}\right]=\bar{\nabla }F_{obs}\left[\xi_{k}\right]+\lambda \bar{\nabla }F_{smooth}\left[\xi_{k}\right]$$

where the prior term $\bar{\nabla }F_{smooth}\left[\xi \right]$ minimizes dynamical amount and is assumed as independent with environment from iteration to iteration.

$$\bar{\nabla }F_{smooth}\left[\xi \right]=\frac{1}{2(n+1)}\sum_{t=1}^{n+1}{∥\frac{q_{t+1}-q_{t}}{\Delta t}∥}^{2}$$

The formulation is stated as finite differencing sense and can be rewrite as

$$\bar{\nabla }F_{smooth}\left[\xi \right]=\frac{1}{2}{∥K\xi +e∥}^{2}=\frac{1}{2}\xi^{T}A\xi +\xi^{T}b+c$$

with a pertinent finite differencing matrix $K=\left[\begin{array}{cccccc} 1 & 0 & 0 & \ldots & 0 & 0\\ -1 & 1 & 0 & \ldots & 0 & 0\\ 0 & -1 & 1 & \ldots & 0 & 0\\ \vdots & \vdots & \vdots & \ddots & \vdots & \vdots \\ 0 & 0 & 0 & \ldots & -1 & 1\\ 0 & 0 & 0 & \ldots & 0 & -1 \end{array}\right]⊗I_{\left(n+2)\times (n+2\right)}$, a constant vector $e={\left[-q_{0}^{T},0,\ldots ,0,q_{n+1}^{T}\right]}^{T}$ governed by boundary conditions $q_{0}$ and $q_{n+1}$, as well as a constant $c=\frac{e^{T}e}{2}$. Accordingly, the acceleration and velocity amount are quantified via Hessian as mentioned above $A=K^{T}K$ and gradient $b=Ka^{T}e$.

Alternatively, the obstacle avoidance gradient $\bar{\nabla }F_{obs}\left[\xi \right]$ prevents per collision checking element from nearing obstacles, or already in a collision.

(10) $$\bar{\nabla }F_{obs}\left[\xi \right]=\sum_{i=1}^{C}J_{c_{i}}^{T}∥X^{\prime }∥\left[\left(I-{\hat{X}}^{\prime }{\hat{X}}^{\prime T}\right)\nabla u-uK\right]$$

in which $J_{c_{i}}$ indicates the corresponding position Jacobian matrix mapping the robot configuration q∈ξ to the position of collision checking element $c_{i}$, which is computed in Equaption (4) and finally sums their total quantity C, as well as $K={∥X^{\prime }∥}^{-2}\left(I-{\hat{X}}^{\prime }{\hat{X}}^{\prime T}\right)X^{\prime \prime }$, denotes curvature vector along the workspace trajectory tracked by a collision checking element. $X^{\prime }$ and $X^{\prime \prime }$ signify the velocity and acceleration of collision checking element, ${\hat{X}}^{\prime }$ expresses the normalized velocity vector. To maintain the workspace gradient as an update direction, the matrix $I-{\hat{X}}^{\prime }{\hat{X}}^{\prime T}$ projects workspace gradient orthogonally to the motion orientation, averting direct influence on the speed profile of trajectory.

### 5.2. Robot Workspace Constraints

To make the optimization query manageable, we need to develop a profitable constraint about robot model. In practice, let the kinematic measurement $f\left(q_{j},c\right)\;:\;R^{3}\in O$ quantity the collision checking elements c on bounding box O, given robot configuration $q_{j}$. The body and leg workspace account for the robot deformable criterion limits and are determined by inverse kinematics of Stewart structure. The specific definition and description has been illustrated in Reference [33]. However, a trajectory ξ is collision-free if the distance from any collision checking point $c_{i}$ to any obstacle is longer than default threshold ϵ>0 and satisfies the case where each robot configuration belongs to the trajectory ($q_{j}\in \xi$).

**Figure 6:**
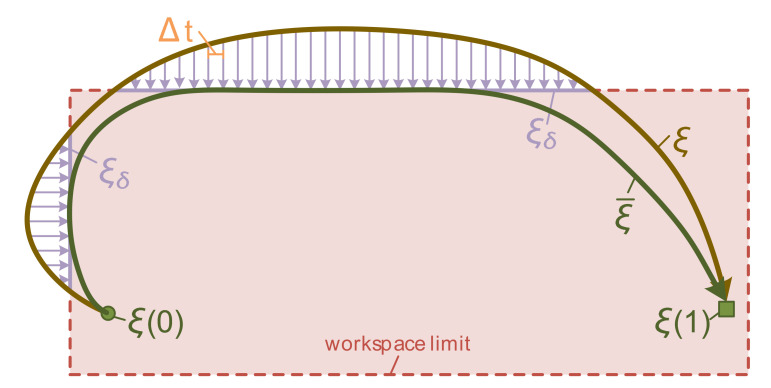
The smooth projection technology for workspace limit demonstrated in two-dimensional plane.

A practical circumstance is a fact that after the updating course illustrated in Section 5.1, much or less part of trajectory configuration might exceed the robot workspace limit even though conducted planning happens in confined space. When all of configurations are optimized for body workspace, the trajectory generation method maintains footholds contacting with ground that is indispensable. With this prerequisite condition, reachable zone of Stewart wheel-leg determines body workspace limit. For the initial purpose of sustaining the trajectory smoothness, one ideal scheme is to create a projection $\xi_{\delta }$ onto the collection of reachable workspace once a workspace violation is recognized. We enforce the approximated projection technique applying the Riemannian metric ${\tilde{\xi }}_{\delta }=A^{-1}\xi_{\delta }$, where $\xi_{\delta }$ is equipped with the most significant feasible boundary of workspace shown in Figure 6. Simultaneously, eliminate workspace violation through measuring value α such that

$$\bar{\xi }=\xi +\alpha {\tilde{\xi }}_{\delta }$$

attains the future optimized trajectory $\bar{\xi }$, until it is entirely inside the workspace limit or iterated to a maximum number times.

Furthermore, it is necessary for robot to achieve a smooth transition between two sequential body configuration within one time segment, avoiding acceleration generation. We specify a maximum step interval constraint $s_{max}$ which is described through euclidean distance to ensure the transition.

### 5.3. Inverse Kinematic Controller

The wheel-quadruped robot can be regarded as a floating-base system, in which the state variables $x={\left[p_{B}^{T}\;\theta_{B}^{T}\right]}^{T}\in R^{6\times 1}$ denote the position and Euler angle attitude of robot body with respect to body frame B. The velocity vector of $i_{th}$ wheel-leg end-effector in map frame M can be written as follows.

(11) $${}^{M}{\dot{F}}_{i}={}^{M}{\dot{p}}_{B}+\omega \times {}^{M}R_{B}\left({}^{B}{\dot{F}}_{i}+{}^{B}J_{i}{\dot{l}}_{i}\right),$$

where ω denotes angular speed of robot body in map frame M, ${}^{B}J_{i}$ represents Jacobian mapping from $i_{th}$ foot point to corresponding joint variables with respect to body frame B. $l_{i}\in R^{6\times 1}$ is the actuator position inputs for $i_{th}$ Stewart mechanism wheel-leg. According to theorem of cross product $\omega \times {}^{M}{\dot{F}}_{i}=-{}^{M}{\dot{F}}_{i}\times \omega$ and ${}^{M}J_{i}={}^{M}R_{B}{}^{B}J_{i}$, we have

(12) $${\dot{F}}_{i}={\dot{p}}_{B}-S\left({}^{M}{\dot{F}}_{i}\right)\omega +{}^{M}J_{i}{\dot{l}}_{i},$$

in which ${}^{M}{\dot{F}}_{i}\times =S\left({}^{M}{\dot{F}}_{i}\right)$ is skew-symmetric matrix. Hereafter, with describing compound motion of robot body and actuators, Equation (12) can be rewritten as matrix formulation that regards I as a unit matrix.

(13) $${\dot{F}}_{i}=\left[I\;-S\left({}^{M}{\dot{F}}_{i}\right)\;{}^{M}J_{i}\right]\left[\begin{array}{c} \dot{x}\\ {\dot{l}}_{i}. \end{array}\right]$$

Let $J_{i}=\left[I\;-S\left({}^{M}{\dot{F}}_{i}\right)\;{}^{M}J_{i}\right]$, this equation becomes

(14) $${\dot{F}}_{i}=J_{i}\left[\begin{array}{c} \dot{x}\\ {\dot{l}}_{i}. \end{array}\right]$$

Calculate the corresponding motion of robot in map coordinate frame M through full-rank pseudo-inverse of $J_{i}$ that is $J_{i}^{+}=J^{T}{\left(JJ^{T}\right)}^{-1}$.

(15) $$\left[\begin{array}{c} \dot{x}\\ {\dot{l}}_{i} \end{array}\right]=J_{i}^{+}{\dot{F}}_{i}.$$

Due to contacting with ground of wheel-leg while robot is rolling through confined space, we can compute actuator trajectories of each electrical cylinder via robot body’s motion.

(16) $${\dot{l}}_{i}={}^{M}J_{i}^{-1}\left({\dot{p}}_{B}-S\left({}^{M}{\dot{F}}_{i}\right)\omega \right).$$

## 6. Results and Discussions

This section represents and discusses the simulation and experiment results based on the entire planning framework shown in Figure 3. Simulations are applied on a simulated BIT-NAZA robot model in Virtual Robot Experimentation Platform (V-REP) furnishing with VORTEX dynamic engine. The BIT-NAZA robot described in Section 2 is used for the experiment verification. Our ultimate prospect is to plan a set of trajectories for COM and deformable footholds polygon from the current configuration to a requested waypoint and traverse the obstacles in various challenging exercises. Four sorts of essential implements endeavor to examine whether the motion generated from the proposed framework is feasible, containing broaden accommodation, lift overhang, rotating clearance, slim gap assignments. All of the planning trajectories were created on the basis of SDF representation of obstacle produced by PCD and obstacle box dimension. We underline that each motion appeared deformably credits to assigned waypoints, user-input velocity, and situational requirement. It also showcased that further guidance in the form of how our method can solve analogical issues with remarkably better terrain-adaptive performance and computational efficiency assessment.

**Figure 7:**
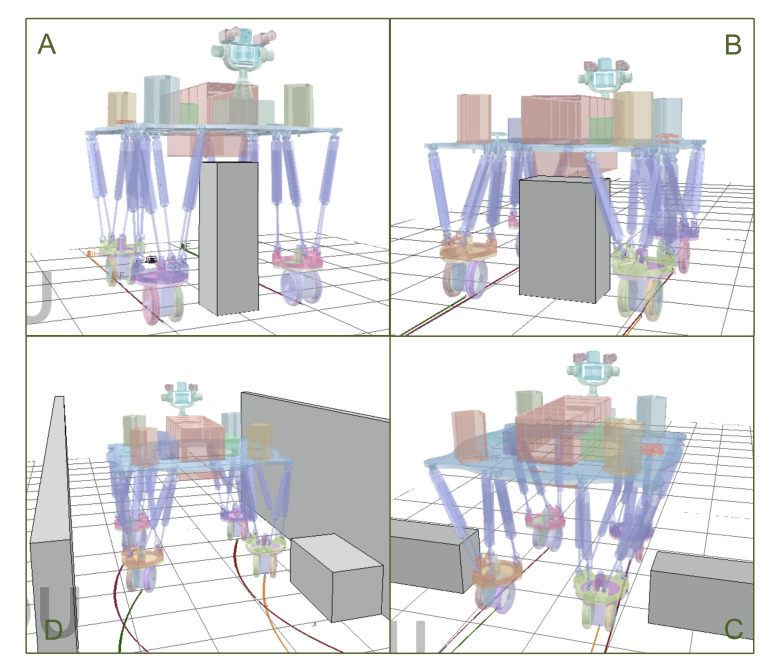
Four assignments to default destination in V-REP simulation: (**A**) Lift overhang routine (**B**) Broaden accommodation routine (**C**) Rotate clearance routine (**D**) Slim gap routine.

Progressively, Figure 7 depicts the produced motion that deformably performs the negotiating capability in four diverse assignments. Each task starts from the neutral configuration when all of the electronic cylinders extend to half of their maximum length as a preparation to willingly shrink and expand. For lift overhang, the robot has to lift its trunk, which results in a corresponding increment in width. We achieved this performance through coupling iterating update Equation (8) with bounding box abstraction through Jacobian $\frac{\partial }{\partial z}c_{j}^{B}$, which was notably mapped to the changes over Z-position *z* that grows. Briefly, the selecting treatment of initial trajectory $\xi_{0}$ was stochastic and even already collides with some obstacles. $\bar{\nabla }F_{obs}\left[\xi_{i}\right]$ and stored SDF prevent bounding box from obstacles and then proceed with numerical optimization. Broaden accommodation event enables the robot to broaden its span and increase the width of bounding box, as evidenced by the linear implementation of $\frac{\partial }{\partial e}c_{i}^{B}$. Slim gap function allows the robot to shrink the width of the bounding box around the body, which is equivalent to that of foothold polygon and passes over the narrow lane, also motivating the differential application of $\frac{\partial }{\partial e}c_{i}^{B}$. Rotate clearance behavior is an extraordinarily non-deformable action that requires the robot to orient its wheels and detour obstacle in a narrow passage. The configurational parameter edits, the steering angle of wheels θ(t), are regulated by synergistic trajectory command in gradient descent update. We note that to obtain these motions, it is necessary to define an input moving velocity 0.1 m/s for active wheels from user manual law. To diminish the frictional error from contacting wheels with the ground and possible slippage error from high dynamic wheeled turnings, mastering adequately low wheeled speed features of great concern.

### 6.1. Simulations

The simulation experiments can help us to analyze and compare the result of the proposed algorithm and robot configuration variation, which is evaluated in 10 trials for each circumstance and obstacle event. In the first lift overhang case (Figure 7A), we progressively change the height of obstacle with increments of 5 cm additional passable height while rolling with neutral configuration. Each time it enables robot to lift accommodate space to pass through obstacle. We found robot could pass over 90 cm obstacle with 100% success rate and the supreme 105 cm with 40%.

During the broaden accommodation event (Figure 7B) in simulation, broadening wheelbase to accommodate wider obstacle rather than bypassing is a better approach to enhance motion efficiency. We examined this case on obstacles with different width and found that robot can pass a 62 cm width box with 100% success and 70 cm with 70% success separately in 10 trials.

Subsequently in the third simulation (Figure 7C), we gradually shrink the gap breadth with reduction of 10 cm to test robot. It is demonstrated that robot could get through a 1.2 m gap with 100% success, 1.1 m with 80%, and 1 m with 30%.

The *rotate clearance* in (Figure 7D) steers robot to change wheel orientation without altering configuration, avoiding obstacle. How much the the maximum turn angle is relies on distance between obstacle and robot. We fixed obstacle on a spot 1 m far from robot and changed different goal to evaluate the turning angle that is 90${}^{\circ }$ with 100% success.

**Figure 8:**
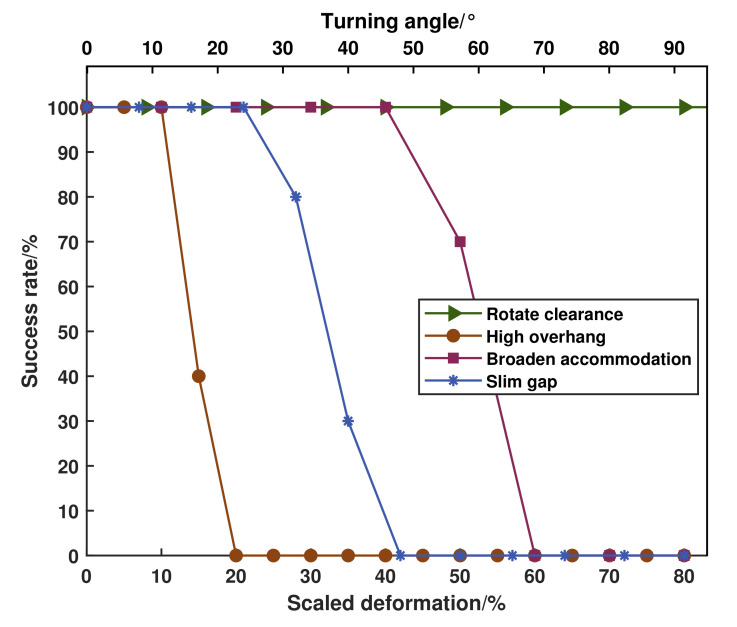
Success rates of increasingly conditional restraints over 10 trials, which are verified in simulation for four specific events.

Although the success rate shown in Figure 8 described the performance of our planning approach, the results may change when we select different parameters. There are many user-defined parameters in the optimization process, such as the learning rate ∇ and the default obstacle threshold ϵ. Larger learning rate can obtain larger trajectory update velocity, boosting sampling interval for next trajectory. But it could miss the local minima point, resulting in a smaller objective function point. Enlarging default obstacle threshold can increase obstacle avoidance ability that prevents trajectory farther from obstacle, but exploring an optimal trajectory in extremely confined space becomes difficult. In our method, the user-defined parameters tuning method generally is based on our experiences or prior knowledge. For example, when optimization fails, we will try to reduce the learning rate. In our future work, we will attempt to apply learning-based methods to select and optimize those parameters intelligently.

### 6.2. Performance Evaluation

For the purpose of graphically visualizing the comparison for deformable adaptation percentage among four mentioned assignments, and we analyze it in simulated cases for security. Furthermore, we examined the success rate of the proposed planner and recognized that it is precisely the absence of diverse environment restriction influences the success rate. 10 simulated examinations were governed for each specific event and condition, whose results are depicted in Figure 8. Only the examining condition for *rotate clearance* case is rotating angle, while the ones for the other three are scaled deformation percentage defined in Section 6.1, which equals to the maximum deformable measurement as a percentage of original volume. Obviously, the maximum steerable angle $\pm 90^{{}^{\circ}}$ evaluated by Stewart kinematics, the success rate for all of rotatable angle in testing *rotate clearance* task reached 100%. Commonly, the success rate of deformable planning algorithm reaches 100% when conditional constraints of target terrain templates satisfied our model abstraction limits, and its variation tendency matches and are smaller than the deformable scale we calculated through kinematic analysis in accordance with Reference [33]. For instance in *lift overhang*, the robot body can be lifted maximum 15 cm that equals to 15% of robot’s neutral height, but the success rate of corresponding environment for robot to attain this configuration is only 40%.

### 6.3. Experiments

**Figure 9:**
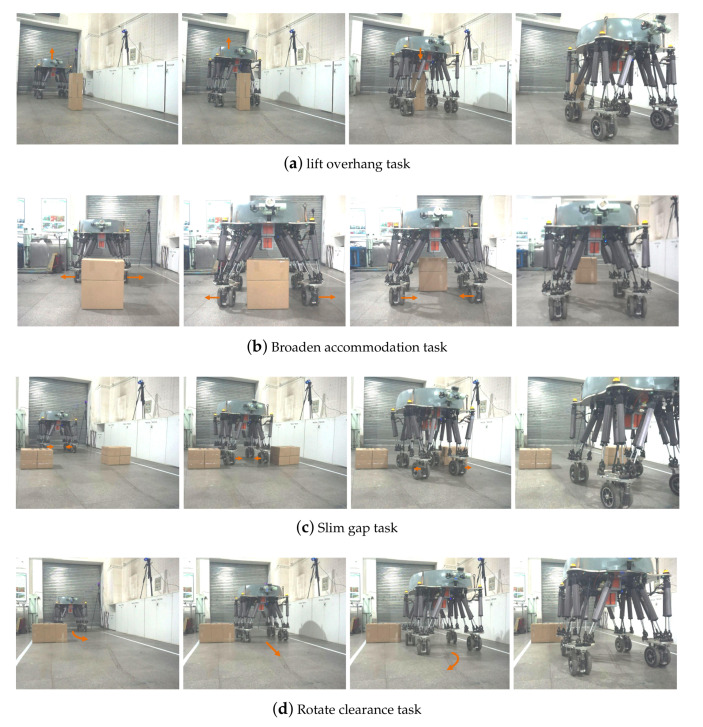
Realistic experiments of four tasks conducting on BIT-NAZA robot.

In addition to experiments, we implemented the entire pipeline from Figure 3 on the BIT-NAZA WLHR such that they demonstrate the deformable capability. Likewise, all of the obstacle-negotiation tasks in a confined space were also performed in realistic situations, as disclosed in Figure 9. The robot detected obstacle locations ${}^{S}P_{i}$ in sensor frame and transformed it together with robot configuration on uniform map frame M, which is defined though the projection of initial COM position and foot contacting surface. The robot posture can be measured by IMU and height can be computed through basic mechanism size and Stewart forward kinematics through extending amount of electrical cylinders. In each experiment, we generated a SDF description around robot with 6 m × 4 m × 3 m dimension scanned from Velodyne-16 LIDAR, which has been elaborated in Section 4. To record the final trajectories generated with four wheels and COG of the robot, we take advantage of displacement sensors embedded in each electrical cylinder. They allow Newton-Raphson iteration of forward kinematics to calculate lateral, altitude trajectories and rotation angles of four Stewart mechanism. The wheeled odometry accumulates and averages the traveled distance of whole robot, that is COM. Apart from the environment perception sensor that VLP-16 LIDAR, we employed an additional camera jointly installed on the controllable holder, identifying the white color line on the ground. Especially in *rotate clearance* assignment, stamping white lines adhered to the ground as 1.6 m-high walls which generates a cluster of corresponding SDF, as a result of avoiding superfluous scene construction. Due to the field size constraint, we only can test each task for one way planning and all of goals are about 3 m far away from COM.

**Figure 10:**
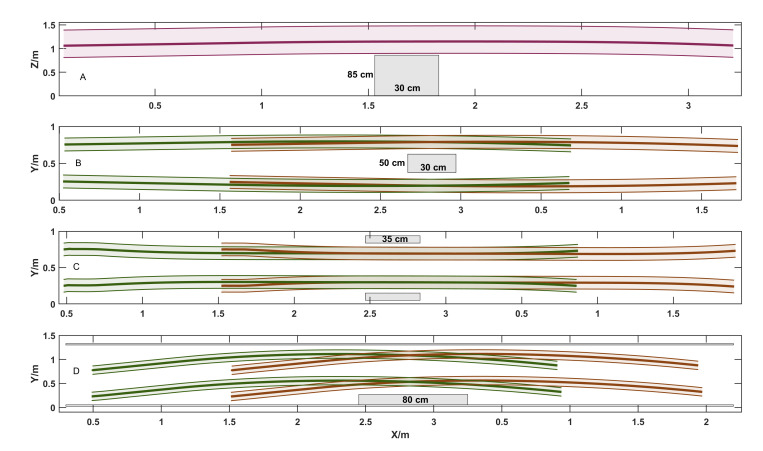
Experiment results from four deformable planning and collision avoidance tasks in confined space.

Note that we utilized the RGB color convention to draw diverse action units and their relationship with obstacles in Figure 10, demonstrating the executive smooth trajectories. The thick brown and green line fractions represent front and hind wheeled trajectories separately, whose corresponding thin lines and enclosed shadow area indicate their dimensional boundary of wheels. The thick strawberry line describes the COM trajectory, how the truck border moves were also portrayed in the same color. Besides, the gray shadow boxes present obstacles in various sizes and scenes. It is sufficiently apparent that robot could negotiate different obstacles and reach the user-specified target waypoints, while deformable iterations optimized the entire motion generation in four experimental cases.

While conducting the first case (Figure 9a), robot encountered an obstacle which had increments of 9 cm additional passable height while rolling with neutral configuration, that was a totally 1 m high box. The goal of the *lift overhang* task is to enable the robot to explore the clearing space upwards, finally recovering to its initial arrangement.

Over the broaden accommodation task (Figure 9b), the robot spanned about 7 cm and 12 cm towards left and right side independently for the obstacle 2.4 m far away. The left and right wheels turn towards outside to enlarge the support polygon which assists robot with the deformable task of accommodating relatively wider obstacle beneath its trunk.

Whereas (Figure 9c) constructed by two obstacles with close distance, the execution of *slim gap* routine demonstrated the adaptive capability to a narrow passing space which possessed the width of 1.3 m. The foothold support polygon shrinked about 12 cm and 9 cm towards left and right respectively. As the width of the bounding box determines the lateral shrinkage, the lateral displacements for front and hind wheels which are on the same side are the same.

The final case (Figure 9d) performed the *rotate clearance* conduction without a morphological configuration of wheelbase. As expected, the proposed planner instructs robot to rotate and get round the right box, steaming into free space on the left side. The left and right walls are too high to cross, so it is impotent to conduct *lift overhang* behavior and reasonably mastery the robot in a confined space. The dimension of the obstacle are length of 0.8 m and width of 0.43 m.

**Figure 11:**
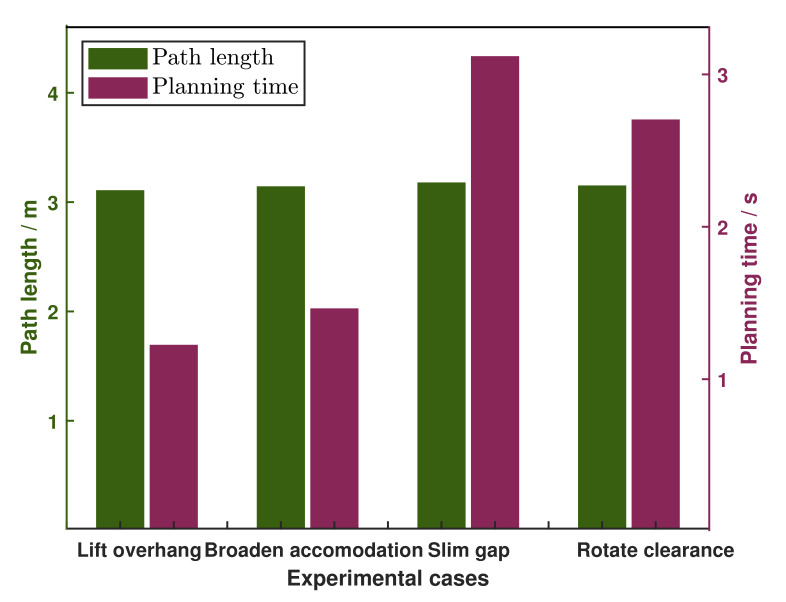
Planning profile plots for four experimental assignments.

Besides, we examine and compare the numerical traits of path length and planning time for four diverse simulated trials, whose results are illustrated in Figure 11. In general, we choose a goal which is about 3 m far from current robot configuration and explore the target trajectory. Although the path lengths for four trials were almost the same, the planning time displayed irregularly in which the one for *slim gap* event was the supreme example. The quantity and intensity of obstacles, as well as unoccupied space in narrow environment directly influence the collision checking efficiency and planning time.

Moreover, it can be carefully scrutinized that after traversed obstacle, the planner had been continuing to gradually lift the robot to extend the trajectory, which picked *lift overhang* behavior as an instance. The appearance of this phenomenon denotes that covariant gradients optimization undertakes searching trajectory with the lowest objective functional metric $\bar{\nabla }U$ where obstacles end up inside current examined coverage between collision checking points. This developed into a trade-off among weighted parts $\bar{\nabla }F_{obs}$ and $\bar{\nabla }F_{smooth}$, since simply adjusting $\bar{\nabla }F_{obs}$ higher than $\bar{\nabla }F_{smooth}$ could cause a more significant possibility of collisions.

## 7. Conclusions

To obtain a versatile trajectory optimization formulation for WLHR, we presented a novel pipeline system based on point cloud data to negotiate obstacle in confined space. With the omnidirectional rolling ability, the wheel-legged hybrid locomotion further exhibited how to leverage a deformable bounding box to abstract the robot model and significantly simplified calculation complexity while merging into the planner. Given PCD around the robot, we developed a suite of obstacle-representing approach that pre-create SDF to store before optimization. This allows our planner to interactively produce deformable trajectories when robot desires to reach requested waypoints. Four assignments that contain *lift overhang*, *broaden accommodation*, *slip gap*, and *rotate clearance* are consequently conducted as compelling examinations for the proposed framework. The simulation analyzed its success rate and limitation for different circumstance. In conclusion, experiments demonstrated the feasibility of our deformable adaptation technique.

The proposed approach manages deformable motion planning and intensifies moving dexterity of wheel-legged robot. Compared to common wheeled robot with fixed configuration, our framework enables robot to go through narrow spaces and roll along a shorter path when confronts with a obstacle.

## Abbreviations

The following abbreviations are used in this manuscript:

COM	Center of Mass
